# Habitat Fragmentation Reduces Genetic Diversity and Connectivity of the Mexican Spotted Owl: A Simulation Study Using Empirical Resistance Models

**DOI:** 10.3390/genes9080403

**Published:** 2018-08-10

**Authors:** Ho Yi Wan, Samuel A. Cushman, Joseph L. Ganey

**Affiliations:** 1 School of Earth Sciences and Environmental Sustainability, Northern Arizona University, Flagstaff, AZ 86011, USA; 2 USDA Forest Service Rocky Mountain Research Station, 2500 S. Pine Knoll, Flagstaff, AZ 86001, USA; scushman@fs.fed.us (S.A.C.); jganey@fs.fed.us (J.L.G.)

**Keywords:** biodiversity, CDPOP, connectivity, endangered species, fragmentation, gene flow, landscape genetics, multiscale, resistance, simulation

## Abstract

We evaluated how differences between two empirical resistance models for the same geographic area affected predictions of gene flow processes and genetic diversity for the Mexican spotted owl (*Strix occidentalis lucida*). The two resistance models represented the landscape under low- and high-fragmentation parameters. Under low fragmentation, the landscape had larger but highly concentrated habitat patches, whereas under high fragmentation, the landscape had smaller habitat patches that scattered across a broader area. Overall habitat amount differed little between resistance models. We tested eight scenarios reflecting a factorial design of three factors: resistance model (low vs. high fragmentation), isolation hypothesis (isolation-by-distance, IBD, vs. isolation-by-resistance, IBR), and dispersal limit of species (200 km vs. 300 km). Higher dispersal limit generally had a positive but small influence on genetic diversity. Genetic distance increased with both geographic distance and landscape resistance, but landscape resistance displayed a stronger influence. Connectivity was positively related to genetic diversity under IBR but was less important under IBD. Fragmentation had a strong negative influence on the spatial patterns of genetic diversity and effective population size (Ns). Despite habitats being more concentrated and less widely distributed, the low-fragmentation landscape had greater genetic diversity than the high-fragmentation landscape, suggesting that highly concentrated but larger habitat patches may provide a genetic refuge for the Mexican spotted owl.

## 1. Introduction

Globally, human-induced environmental change degrades habitats and drives biodiversity loss and species extinctions across nearly all taxonomic groups [1,2,3]. Threatened and endangered species are particularly vulnerable to habitat loss and degradation. The 2015 International Union for Conservation of Nature (IUCN) Red List identifies habitat loss and degradation as the main threat to 85% of all 79,837 species being assessed [4]. Habitat loss and fragmentation increase spatial isolation of populations, reduce population size, and disrupt dispersal behavior and population connectivity [5,6], leading to potential reduction in gene flow and subsequent decline in genetic diversity [7,8,9]. In this context, habitats that provide connectivity and linkages for dispersal and gene flow have high conservation value, especially in fragmented landscapes. In response to growing concerns about habitat loss and fragmentation, the identification and protection of wildlife corridors has become an important tool for conserving landscape connectivity and gene flow of rare species [10,11]. Meanwhile, distribution and genetic data on rare species are often sparse because of small population size and low detection probability, and existing data are usually restricted to small or local regions. As a result, identifying important areas for conserving connectivity and gene flow over large landscapes for rare species can be challenging.

Landscape genetics is an emerging discipline that integrates landscape ecology and population genetics to statistically analyze the relationship between population genetic structures and landscape features [12,13]. The emergence of landscape genetics has given rise to new and promising analytical methods to explicitly quantify the effects of landscape heterogeneity on spatial patterns of genetic processes, such as gene flow, genetic drift, and adaptation [14,15,16,17]. One of these innovations is the integration of connectivity modeling and individual-based population genetics simulation for determining broad-scale genetic connectivity patterns [18,19]. Simulation studies provide a feasible solution for evaluating spatial genetic structure and differentiation when empirical genetic data for target species are scarce or unavailable (e.g., [20,21]). These methods have been increasingly used to aid the identification and design of corridors that maintain and restore linkages between habitat patches and facilitate biotic processes such as dispersal and gene flow across many taxa, including mammals [22,23,24], fish [25], insects [26], and plants [18,27].

Isolation-by-distance (IBD) is a common null model in landscape genetics that addresses whether genetic differentiation exists among populations as a function of geographic distances alone. IBD, however, does not consider the effects of heterogeneity in landscape features. One common approach that evaluates the effects of landscape heterogeneity on genetic connectivity is isolation-by-resistance (IBR) modeling [28,29,30], which quantifies the extent to which landscape features act as resistance to movement of genes.

Although IBR may complement IBD to provide a more complete depiction of relationships between landscape features and spatial population processes, its predictions are sensitive to the assumptions, methods, and data sources of landscape resistance models. Quite often, multiple resistance models derived from different data sources and geographic areas are available for the same species. These models often depict different spatial configurations of habitat extensiveness and fragmentation, which strongly affect detectability of landscape genetics relationships [31]. The validity of these models within a different spatial or temporal space is unknown until tested. Hence, it is important to consider different possibilities by comparing model predictions. A meta-analysis that combines multiple resistance models can be useful for detecting the true effects of landscape patterns, such as fragmentation, on genetic processes.

According to a recent review, birds are the most underrepresented taxa in the landscape genetics literature, and most bird species in these studies have relatively low dispersal ability [32]. Interestingly, birds have traditionally benefited from taxonomic bias and have been overrepresented in ecology and conservation research compared to other taxa [33,34,35,36,37,38]. Such contrasting taxonomical bias in landscape genetics may be due to the presumed high vagility and low spatial genetic variability of birds relative to other taxa [32], notwithstanding the fact that bird species, even those with long dispersal distance, can exhibit spatial genetic variation [39,40,41]. There is a critical need for landscape genetics studies on bird species to improve the understanding of potential drivers that shape their spatial genetic structures.

In this study, we sought to understand how differences between landscape resistance models might affect predictions of gene flow processes and genetic diversity for a rare bird species of high conservation importance, the Mexican spotted owl (*Strix occidentalis lucida*). This owl is highly selective for nesting habitats and the availability of those habitats appears to limit its distribution [42]. At the same time, it inhabits landscapes in which the distribution of those nesting habitats is naturally fragmented [42] and is capable of dispersing over long distances and through habitats that differ greatly from nesting habitat [43,44,45,46]. Thus, despite the strong selection for particular types of nesting habitat, it may be less sensitive to habitat fragmentation than many other studied species. This owl provides an interesting model species in this context.

To evaluate differences between landscape resistance models, we simulated the gene flow and population dynamics of the Mexican spotted owl using two empirical landscape resistance models applied to the same geographic area, resulting in different degrees of fragmentation as well as a null model of IBD. The two resistance models were developed based on two habitat suitability models that showed strong performance [47,48], representing two reasonable scenarios of how the Mexican spotted owl perceived the landscape. We simulated individual-based breeding and dispersal movements and analyzed a suite of genetic diversity metrics in conjunction with connectivity models developed from previous works [47,48] to evaluate: (1) the effect of landscape connectivity and fragmentation on spatial population patterns; (2) genetic divergence as a function of geographical and resistance distance; (3) genetic diversity as a function of landscape connectivity; and (4) how genetic diversity and divergence might differ between the two landscape resistance models examined.

## 2. Materials and Methods

### 2.1. Study Species

The Mexican spotted owl is one of three recognized spotted owl subspecies in North America. It typically occurs in forested habitats with high canopy cover of mixed-conifer or pine-oak [49,50,51,52,53] but also occurs in rocky canyonlands [54]. Nest and roost sites are mostly found in deep, narrow canyons and on steep slopes [42,55,56]. These habitats are widespread but naturally fragmented throughout the southwestern United States and Mexico, so populations of the Mexican spotted owl are patchily distributed. The range of the Mexican spotted owl extends from Utah and Colorado through Arizona, New Mexico, and western Texas to southern Mexico. The Mexican spotted owl has experienced population decline [57,58] primarily due to habitat loss and fragmentation from logging [42]. Although the Mexican spotted owl is now protected by the Endangered Species Act as a Threatened species [59], its recovery is shrouded by uncertainty due to emerging threats such as uncharacteristically large and severe wildfires and climate change, which have progressively become more influential in the southwestern United States and have the potential to cause large-extent habitat reduction [42,60,61].

### 2.2. Study Area

**Figure 1:**
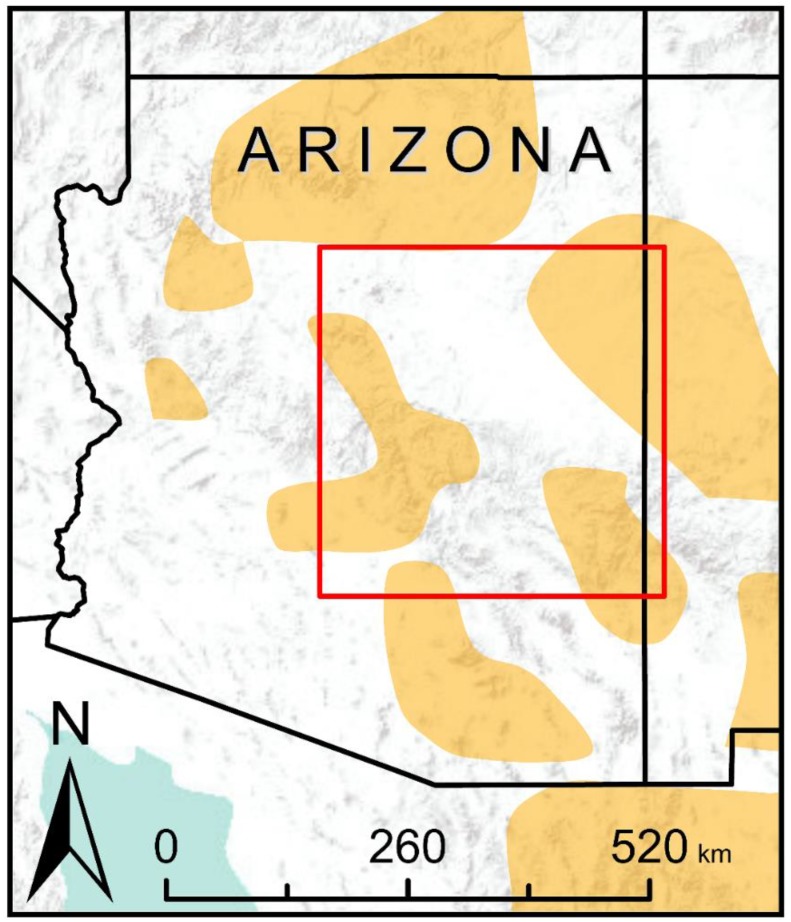
Red box indicates the boundary of the study area. Spatial extent is identical to the study area of Wan et al. [48] for direct comparative analyses. Orange polygons indicate the general range of the Mexican spotted owl.

Our study area was identical to the study area of Wan et al. [48], covering an extensive area of northern Arizona, USA (latitude 32.6–35.4° N, longitude 108.6–112.1° W; Figure 1). We selected this study area for three reasons. First, it allowed us to use habitat suitability, resistance, and connectivity models produced by Wan et al. [48] for conducting our simulations and statistical analyses. Second, habitat amount and fragmentation varied spatially across this area, providing interesting variation both within and between models. Third, this area included part of what is considered the core range of the Mexican spotted owl [42], making the results of high conservation interest. 

### 2.3. Resistance Models

To evaluate how differences in landscape resistance models might affect gene flow, we used two resistance surfaces developed by Wan et al. [48] in our landscape genetics simulations. The two resistance surfaces were derived from negative exponential transformation (e.g., [62]) of two strong habitat suitability models (Area Under the Curve = 0.91 and 0.88, respectively) [48]. The underlying habitat models were developed with the same multiscale optimization method [63] but were applied to two independent owl datasets collected from within a portion of our study area [55] and approximately 400 km from our study area [56]. The habitat models contained many of the same variables that were important to the Mexican spotted owl, which generally described topography or habitat composition, but the relative importance of individual variables differed between the models, with topographic variables dominating one model [55] and composition variables dominating the other [55,56]. 

The resistance surfaces had values between 1 and 10 at any given pixel (pixel size = 30 × 30 m), representing the relative permeability of landscape features to species movement. A high resistance value (near 10) indicated areas the species was unlikely to traverse, and a low resistance value (near 1) indicated areas the species was likely to traverse. Both models predicted fragmented areas of low resistance that were patchily distributed across the study area, but areas of low resistance were more aggregated as larger patches in the first model (hereafter low-fragmentation model), whereas the second model had areas of low resistance that were more fragmented and scattered across linear canyon terrain throughout the study area (hereafter high-fragmentation model). Thus, the two empirically based habitat models produced resistance surfaces that differed in habitat configuration, providing two plausible models describing current habitat connectivity in this area. These differences in connectivity in turn may drive future differences in gene flow across the landscape.

### 2.4. Fragmentation Analysis

To characterize habitat fragmentation, we transformed the probability maps of habitat suitability predicted by the two models into binary maps of habitat versus non-habitat using 0.2 as the cutoff point (i.e., suitability > 0.2 was classified as habitat and suitability < 0.2 was classified as non-habitat). We chose a relatively low value as the cutoff point (i.e., 0.2) because the owl is a mobile species and can disperse through most habitats. Then, we used FRAGSTATS [64] to calculate a suite of landscape metrics, including percentage of landscape (PLAND), mean patch size (AREA_MN), number of patches (NP), patch density (PD), largest patch index (LPI), and radius of gyration (GYRATE; a measurement of correlation length or extensiveness of patches).

### 2.5. Landscape Genetics Simulation

We evaluated patterns of gene flow under two hypotheses:

1. Isolation-by-distance (IBD): This hypothesis serves as a null model and assumes that species movement decisions are affected purely by geographic distance, and genetic exchange occurs more frequently between proximate individuals than distant individuals.
2. Isolation-by-resistance (IBR): This hypothesis posits that species movement decisions and resulting gene flow are influenced by landscape features and patterns associated with resource selection.

For each hypothesis, we used the Cost Distance Populations (CDPOP) simulation model [65] to simulate the processes of breeding and dispersal movement. CDPOP is a spatially explicit, individual-based landscape genetics simulation tool that models population dynamics and genetic exchange as functions of individual-based movement on a resistance surface. For IBD, the resistance surface can be viewed as a uniform raster with a resistance value of 1. For IBR, resistance surfaces described above were used. To represent potentially occupiable locations of individual owls, we used the habitat suitability map developed by Wan et al. [48] as an input probability raster to randomly generate 1000 spatially balanced nodes (i.e., higher-probability areas had more random nodes and lower-probability areas had fewer nodes). Each node could have up to two individuals. We randomly populated 800 nodes (i.e., 80% occupancy), each with 2 individuals, for a total of 1600 initial individuals for the simulations.

**Figure 2:**
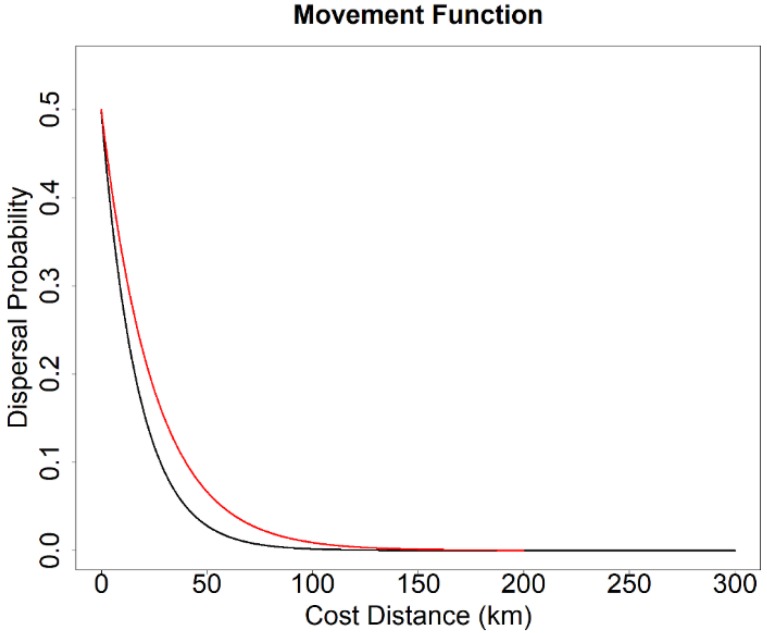
Functions used to transform cost distance to dispersal probability for parameterizing landscape genetics simulations. Black curve represents dispersal probability function of the high-dispersal scenario (300 km). Red curve represents dispersal probability function of the low-dispersal scenario (200 km).

In our simulations, we considered two upper dispersal limits—high dispersal ability (300 km) and low dispersal ability (200 km). We selected these two dispersal limits because they were identical to the dispersal limits in the connectivity models of Wan et al. [48], which enabled us to conduct statistical comparison analyses. The two dispersal limits corresponded to 300,000 and 200,000 cost units on a uniform landscape of a minimum resistance value of 1. Although spotted owls are capable of long-distance dispersal (>400 km) [46,66], most disperse <50 km [44,67,68]. To emulate that, probability of dispersal in the simulation used a negative exponential function of distance such that ~90% dispersal movements were <50 km (Figure 2). 

The model stipulated that individuals would only mate with the nearest neighbor to represent monogamous territorial mating. Fecundity was parameterized to a mean of 3.8 offspring per pair per generation with a Poisson distribution to approximate fecundity over ~5 successful breeding seasons (~0.76 young per pair per year across multiple studies) [69,70,71]. A 50:50 sex ratio was used and there was no difference between male and female dispersal movement. At the beginning of the simulation, each individual was randomly assigned 30 genetic loci, with 10 alleles per locus.

We tested eight scenarios, reflecting a full factorial design of three factors: (1) resistance model (i.e., the low-fragmentation model vs. the high-fragmentation model), (2) isolation hypotheses (i.e., IBD vs. IBR), and (3) maximum dispersal ability (i.e., 200 km vs. 300 km). For each scenario, we performed 100 Monte Carlo simulation runs. Each run simulated dispersal and breeding movements of 100 discrete and nonoverlapping generations. We used nonoverlapping generations to simplify and accelerate the genetic processes in the simulations, which allowed us to project forward into the future with fewer iterations.

### 2.6. Spatial Population Patterns

We tracked the number of extant individuals at each node at the 100th generation of each simulation run and calculated the mean effective population size (N_s_) at each node using R package sGD [72,73]. We conducted moving window analyses with a neighborhood radius of 20 km to map the spatial patterns of population density.

### 2.7. Genetic Diversity

To evaluate genetic diversity, at the end of each simulation run (i.e., the 100th generation), we calculated standard indices including allelic richness (A_r_), observed heterozygosity (H_o_), and expected heterozygosity (H_e_) within each node using R package sGD [72,73]. Then, we calculated the mean indices of all Monte Carlo runs at each node. We conducted moving window analyses with a neighborhood radius of 20 km to create smoothed continuous surfaces of the indices across the study area to map spatial patterns of genetic structures.

### 2.8. Resistance Cost Distance and Genetic Distance

To assess the effects of the two isolation hypotheses on genetic divergence, we calculated pairwise resistance distance and genetic distance between each node. For IBR, resistance distance was quantified with the least-cost path approach, indicating the lowest cumulative path resistance between nodes. For IBD, resistance distance was the total resistance of the least-cost path on a resistance surface in which all pixels were assigned a value of one. Resistance distance was calculated with the *ecodist* package in R [74]. To measure genetic distance, we used the *propShared* function in *adegenet* package in R [75] and calculated the proportion of shared alleles (D_ps_) for each pair of nodes. Note, genetic distance, by definition, is a measure of the dissimilarity as a function of distance, whereas D_ps_ is a measure of similarity. Therefore, D_ps_ and genetic distance are inversely related.

After obtaining the matrices of resistance distance and genetic distance, we conducted two analyses. First, we calculated Mantel correlations [76] between matrices of genetic distance and resistance distance using the *ecodist* package in R [74]. Second, we vectorized the matrices and then fitted linear, logarithmic, and exponential regression models to identify the best fit for depicting genetic distance as a function of resistance distance.

### 2.9. Genetic Diversity and Landscape Connectivity Relationships

We evaluated whether relationships of landscape connectivity and genetic diversity differed between the two resistance models by using the connectivity models of Wan et al. [48]. The connectivity models were developed with the cumulative resistant kernel approach [77] in landscape connectivity simulation software UNICOR [78]. Wan et al. [48] applied resistance surfaces from the low-fragmentation and high-fragmentation models to calculate the cumulative resistant kernel density within a maximum dispersal distance of 200 and 300 km, respectively. The cumulative resistant kernel density is equivalent to the spatial incidence function of the frequency of dispersing individuals found in every cell in the landscape [79] and the value of the cumulative resistant kernel surfaces indicates the expected density of dispersing individuals at any given pixel in our study area [48]. At each node, we extracted the value of the cumulative resistant kernel surface from the connectivity models of Wan et al. [48]. We again fitted linear, logarithmic, and exponential regression models to identify the best fit that relates connectivity strength to the standard indices described above.

## 3. Results

### 3.1. Fragmentation Analysis

**Figure 3:**
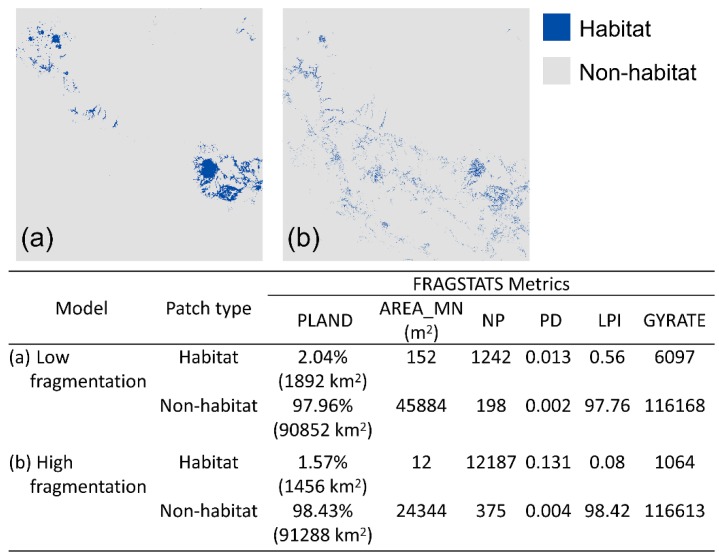
Habitat and non-habitat binary maps predicted by (**a**) low-fragmentation [55] and (**b**) high-fragmentation models [56] using a 0.2 suitability threshold. FRAGSTATS metrics that characterizes fragmentation of habitat and non-habitat patches of the two models are shown in bottom table. PLAND: percentage of landscape, AREA_MN: mean patch size, NP: number of patches, PD: patch density, LPI: largest patch index, and GYRATE: radius of gyration of patches.

Amount of habitat did not differ greatly between our two landscapes, with habitat patches comprising approximately 1.5%–2% of the entire landscape among the two fragmentation models (Figure 3). The high-fragmentation model had a higher number and density of habitat patches than the low-fragmentation model (Figure 3). Largest patch index of habitat patches for the low and the high-fragmentation models were 0.56 (519 km^2^) and 0.08 (74 km^2^), respectively. Mean habitat patch size was larger in the low-fragmentation model than in the high-fragmentation model (Figure 3). Also, habitat patches in the low-fragmentation model were spatially more extensive, as indicated by the higher mean radius of gyration (Figure 3).

### 3.2. Spatial Patterns of Effective Population Size and Genetic Diversity

**Figure 4:**
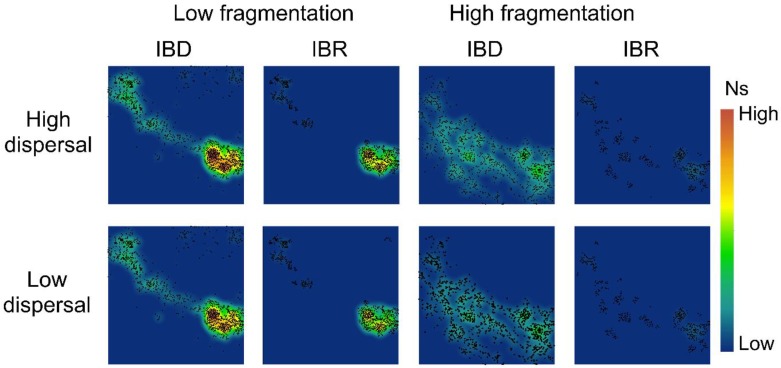
Spatial patterns of effective population size (N_s_) in the low-fragmentation model (left two columns) and the high-fragmentation model (right two columns). IBD: isolation-by-distance. IBR: isolation-by-resistance. Top row: high-dispersal scenario (300 km). Bottom row: low-dispersal scenario (200 km). Black marker represents occupied nodes at the 100th generation of simulation.

**Figure 5:**
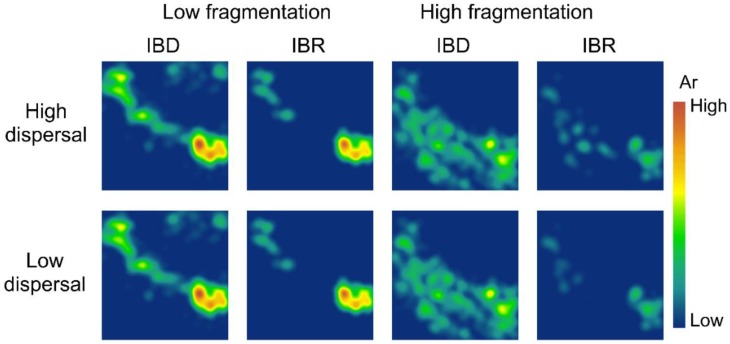
Spatial patterns of allelic richness (A_r_) in the low-fragmentation model (left two columns) and the high-fragmentation model (right two columns). Top row: high-dispersal scenario (300 km). Bottom row: low-dispersal scenario (200 km).

Our simulations showed little difference in spatial genetic patterns between high-dispersal and low-dispersal scenarios (Figure 4 and Figure 5). The low-fragmentation model and the high-fragmentation model produced different spatial patterns of N_s_ and genetic diversity across all indices. For the low-fragmentation model, populations were concentrated as a single cluster in the southeastern parts of the study area under both isolation hypotheses, with a narrow corridor of populations that extended from this cluster to the northeastern parts of the study area (Figure 4). In the high-fragmentation model under IBR, N_s_ was low because of sparse neighborhoods of occupied nodes, whereas under IBD, areas with a moderate level of N_s_ (i.e., ~20) were patchily distributed throughout the southern half of the study area (Figure 4). Thus, the spatial pattern of mean population size differed under each of the two spatial models.

For genetic diversity, under IBD with the low-fragmentation model, the spatial patterns of genetic diversity were similar but with small clusters of islands that formed a stepping-stone-like corridor connecting the southeast and northwest (Figure 5, Figures S1 and S2). In the low-fragmentation model under IBR, areas with high genetic diversity were concentrated in the southeastern parts of the study area, and small islands with moderate levels of A_r_, H_o_, and H_e_ were also observed in the northwest of the study area (Figure 5, Figures S1 and S2). The high-fragmentation model predicted overall lower genetic diversity than the low-fragmentation model (Figure 4 and Figure 5). Under this model and IBD, areas with moderate levels of H_o_, H_e_, and A_r_ were few and highly fragmented (Figure 5, Figures S1 and S2). Under IBD, across all genetic indices, areas with a moderate level of diversity were patchily distributed throughout the northwest to southern half of the study area (Figure 5, Figures S1 and S2).

### 3.3. Genetic Distance Increased with Resistance Distance

**Figure 6:**
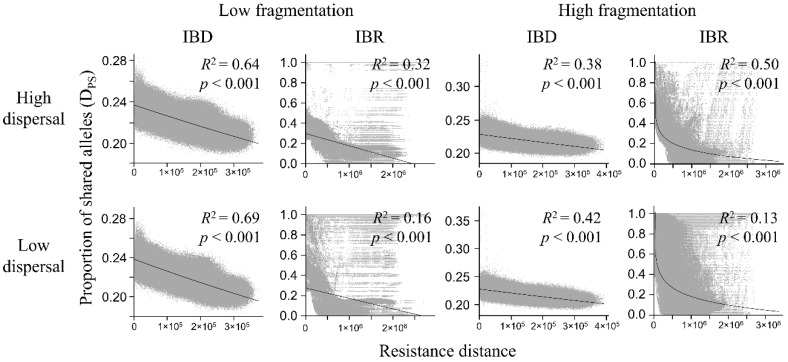
Relationship between pairwise D_ps_ and resistance distance in the low-fragmentation model (left two columns) and the high-fragmentation model (right two columns). Top row: high-dispersal scenario (300 km). Bottom row: low-dispersal scenario (200 km). Proportion of shared alleles (y-axis) is calculated with *ecodist* R package. Resistance distance (x-axis) is the least-cost path resistance between nodes. Gray markers represent each pairwise node distance. Adjusted *R*^2^ and *p*-value are shown.

In both the Mantel test and regression analysis, the D_ps_ was negatively related to resistance distance (Table 1, Figure 6; complete results of regression analyses, including *R*^2^, *p*-value, and the best form of regression (i.e., linear, logarithmic, and exponential), are provided in Tables S1–S5). The strongest negative correlation with resistance distance was in the low-fragmentation model under IBD (Table 1). Under IBR, correlations were significant but weaker. In the high-fragmentation model, correlations between D_ps_ and resistance distance in the high-dispersal scenario were moderately strong under both IBD and IBR. In the low-dispersal scenario, the correlation was also moderately strong under IBD but weak under IBR.

Exponential regression models had the best model fit (i.e., highest *R*^2^) for all IBD models, the linear model had the best fit for the low-fragmentation IBR models, and the logarithmic model had the best fit for the high-fragmentation IBR models (Table S1). Under all scenarios, D_ps_ decreased with resistance distance (Figure 6). The low-fragmentation model under IBD showed the strongest relationship between D_ps_ and resistance distance (Figure 6). Under IBR, the association ranged from moderate to weak. The high-fragmentation model showed a generally weaker relationship between D_ps_ and resistance distance compared to the low-fragmentation model. Under IBD, there was a moderate relationship between D_ps_ and resistance distance. Under IBR, it ranged from moderate to weak.

### 3.4. Population Size and Landscape Connectivity Relationships

**Figure 7:**
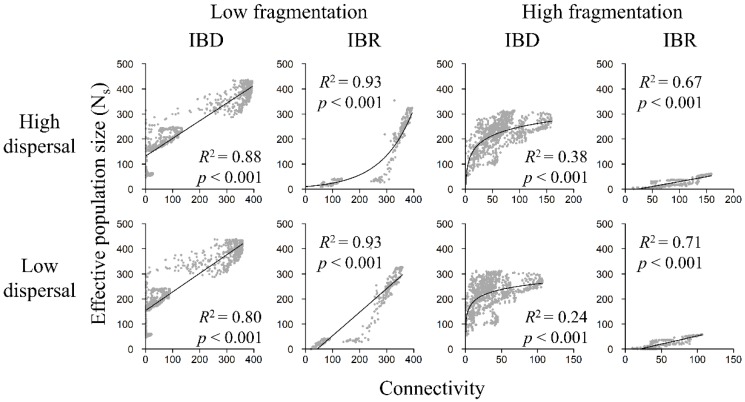
Relationship between N_s_ and landscape connectivity in the low-fragmentation model (left two columns) and the high-fragmentation model (right two columns). Top row: high-dispersal scenario (300 km). Bottom row: low-dispersal scenario (200 km). Effective population size (y-axis) is calculated with software sGD. Connectivity (x-axis) is quantified in terms of cumulative resistant kernel density, representing the expected density of dispersing individuals. Gray markers represent the mean of 100 Monte Carlo simulation runs for each node. Adjusted *R*^2^ and *p*-values are shown.

**Figure 8:**
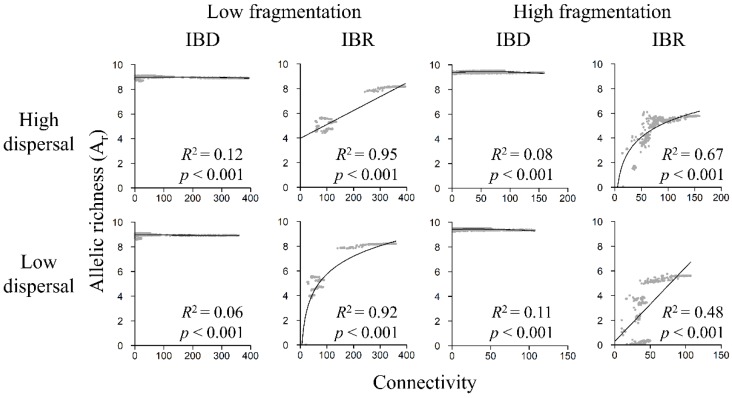
Relationship between A_r_ and landscape connectivity in the low-fragmentation model (left two columns) and the high-fragmentation model (right two columns). Top row: high-dispersal scenario (300 km). Bottom row: low-dispersal scenario (200 km). Allelic richness (y-axis) is calculated with software sGD. Connectivity (x-axis) is quantified in terms of cumulative resistant kernel density, representing the expected density of dispersing individuals. Gray markers represent the mean of 100 Monte Carlo simulation runs for each node. Adjusted *R*^2^ and *p*-value are shown.

N_s_ increased with landscape connectivity in both the low-fragmentation model and the high-fragmentation model and among all scenarios (Figure 7). Regression analyses showed that linear models were best (i.e., highest *R*^2^) at describing relationships between population size and landscape connectivity for most scenarios (Table S2). The logarithmic model was best at depicting N_s_ in the high-fragmentation IBD models. The exponential model only had the highest *R*^2^ in the low-fragmentation IBR model under the high-dispersal scenario. Under IBD, N_s_ showed a strong positive relationship with landscape connectivity in the low-fragmentation model (Figure 7 and Figure 8) but a weaker positive relationship in the high-fragmentation model. Under IBR, N_s_ was strongly and positively related to connectivity among all scenarios.

### 3.5. Genetic Diversity and Landscape Connectivity Relationships

Under IBR, all genetic indices increased with connectivity under both fragmentation models and among both high- and low-dispersal scenarios (Figure 8, Figures S3 and S4), but relationships were weak under IBD. Linear models were best at describing relationships between genetic diversity and connectivity among most scenarios except for the low-fragmentation IBR low-dispersal scenario and the high-fragmentation IBR high-dispersal scenario, in which logarithmic models had better model fit (Tables S3–S5). Under IBR, in the low-fragmentation model, A_r_, H_o_, and H_e_ exhibited strong positive relationships with connectivity (Figure 8, Figures S3 and S4). In the high-fragmentation model, the relationships were also strong under the high-dispersal scenario but weaker under the low-dispersal scenario. Under IBD, A_r_, H_o_, and H_e_ were weakly related to landscape connectivity in the low-fragmentation model (Figure 7 and Figure 8) and the high-fragmentation model (Figure 8, Figures S3 and S4).

## 4. Discussion

We conducted the first landscape genetics study using individual-based simulations to model broad-scale gene flow processes of the Mexican spotted owl in fragmented landscapes. Our results suggested strong differences in future spatial genetic patterns between (1) models that contained similar amounts of habitat but differed in degree of fragmentation of that habitat and (2) hypotheses based on IBD versus IBR. In contrast, our simulations suggested fewer differences in spatial genetic patterns in these landscapes between the two dispersal distances tested. 

Our simulation results indicated that that both geographic distance and landscape resistance increased genetic differentiation among individuals at the regional level (Figure 6, Table 1) but with landscape resistance contributing to larger potential impacts on genetic divergence than did distance alone (Figure 6, Table 1). For example, in the high-dispersal scenario of the low-fragmentation model, average D_ps_ decreased by ~4% between nodes with the greatest distance under IBD (Figure 6), whereas average D_ps_ decreased by ~20% between nodes with the greatest resistance distance under IBR. Also, the importance of geographic distance and landscape resistance varied by the dispersal ability of the species and by the degree of fragmentation on the landscape. For example, geographic distance played a bigger role in the differentiating genetic structures when the dispersal ability of the species was low, especially under a less fragmented landscape (Figure 6). Conversely, landscape resistance increased in importance when the dispersal ability of the species was high and on a highly fragmented landscape (Figure 6).

### 4.1. Connectivity Facilitated Gene Flow

Our results suggested that having greater connectivity between habitat patches could help maintain genetic diversity, although it appeared that the facilitative effect plateaus when connectivity strength reaches a certain “threshold” (Figure 7 and Figure 8). For example, in the low-fragmentation IBR model, genetic diversity (i.e., H_o_, H_e_, and A_r_) began to stabilize when connectivity strength (i.e., x-axes in Figure 7 and Figure 8) reached ~200. The stabilized diversity was similar to potential maximum diversity (i.e., the simulated range of genetic diversity in the null IBD model; y-axes in Figure 8, Figures S3 and S4). We observed a similar threshold effect at connectivity strength of ~50 in the high-fragmentation IBR model. There also appeared to be greater variability when connectivity strength was low because fragmentation limited gene flow and destabilized genetic structures. Cushman et al. conducted a landscape genetics study on simulated landscapes of varying habitat configurations and found that landscape genetic effects might not be detectable when habitats were highly connected because gene flow was not limited by spatial patterns of landscape features [31]. Consistent with their findings, this study shows that, even on landscapes from empirical models, landscape genetic effects are more detectable when connectivity is low and less detectable when connectivity is high and reaches the aforementioned threshold.

Also, the importance of geographic distance and landscape resistance varied by dispersal ability of the species and by the degree of fragmentation on the landscape. For example, geographic distance played a bigger role in the differentiating genetic structures when the dispersal ability of the species was low, especially under a less fragmented landscape (Figure 6). Conversely, landscape resistance increased in importance when the dispersal ability of the species was high and on a highly fragmented landscape (Figure 6). We tested only two dispersal distances, and both allowed for relatively long dispersal distances relative to most distances moved by spotted owls [44,68,80]. Thus, allowed movements may have been relatively liberal relative to the degree of connectivity in our study landscapes, suggesting that we may have minimized our ability to detect spatial patterns and threshold effects on our study landscapes. Arguing against this conclusion, however, note that the negative exponential function used to model dispersal distance guaranteed that most dispersal movements were short (Figure 2). 

### 4.2. Linear, Logarithmic, and Exponential Relationships

Genetic distances often do not form clear linear relationships to landscape distances because of confounding factors and landscape complexity [81,82]. Log-transformed data are sometimes used to improve the linearity of landscape genetic relationships [81,83,84], although strong improvements over untransformed data are not always observed [83,84]. In this study, we used linear, logarithmic, and exponential regression models to account for potential nonlinear relationships. Most relationships were best explained by linear models, but logarithmic and exponential models showed a better fit to the data in some scenarios. In the cases where exponential models were superior to linear models, the improvement was small, however (Table S1). Whereas, when the best fit was logarithmic, the improvement was greater (Tables S1–S5). Ecologically, a logarithmic fit indicates that genetic differentiations can occur quickly when populations are isolated. Conversely, an exponential fit suggests that IBD effects become more detectable when a certain distance threshold has been breached. Other forms of regression models (e.g., inverse regression, nonlinear regression, etc.) are available and are worth further investigation in future studies.

### 4.3. Aggregated and Concentrated vs. Fragmented and Widespread Landscapes

This study sheds light on an interesting question related to the classic SLOSS (single-large or several-small) debate [85,86]: are population size and genetic diversity better preserved on an aggregated but concentrated landscape or on a highly fragmented but widely distributed landscape? Our results appear to support the former because habitat amount differed far less than habitat configuration and dispersion between the low- and high-fragmentation models. For example, many nodes were expected to have an effective population of >100 owls, with some reaching >300 owls, in the low-fragmentation IBR models (Figure 7). In contrast, none of the nodes reached 100 owls in the high-fragmentation IBR models. In addition, a comparison of genetic indices revealed that the low-fragmentation model had overall greater genetic diversity than the high-fragmentation model. For example, H_o_ and H_e_ reached >0.8 in the low fragmentation IBR models but only reached ~0.7 under the best high-fragmentation IBR scenario (Figures S3 and S4). For a threatened species, a 10% decline in heterozygosity might have a lasting negative impact. In the case of this study, fragmentation disrupts population and genetic connectivity, and its negative effects on population size and genetic diversity outweighed the benefits of having broadly distributed (but fragmented) core areas.

The isolation effect on genetic diversity has long been theorized [87] and empirically demonstrated [88,89,90] but has recently been challenged by the habitat amount hypothesis [91]. This hypothesis posits that genetic structure is best predicted by habitat amount, and that habitat configuration such as fragmentation, continuity, and isolation can be ignored. Empirical studies that have examined the relative importance of habitat amount versus habitat configuration have yielded contradictory results, often muddled by inconsistency of landscape and genetic metrics used in the analyses [92]. Simulation studies on this topic have been rare and also yielded inconsistent results (e.g., [93,94,95]). Our results based on empirical resistance models and simulated gene flow suggest that the amount and continuity of habitat are both important for determining genetic diversity, but habitat configuration seems to be more important than the amount because the latter did not vary much overall between the two models. We see an opportunity for more empirical and simulation studies to further understanding on this topic.

### 4.4. Comparison with Empirical Data on Genetic Structure

Few previous genetic studies have featured the Mexican spotted owl and none have explicitly considered the context of landscape features [96,97,98,99,100]. These studies used empirical genetic data, but because the Mexican spotted owl is a rare species, sample sizes in these studies were either small or uneven by region. In the most extensive study in terms of sample size and spatial coverage, Barrowclough et al. [100] concluded that most of the variability in genetic structure across the range of the Mexican spotted owl occurred among distinct geographic regions, with much lower levels of genetic variation observed among populations sampled within those regions. They concluded that gene flow was substantial among populations within the relatively continuous habitat zone of the Mogollon Rim (i.e., the northern portion of our study area). Outside of that region, however, genetic divergence increased exponentially with geographic distance among fragmented populations on the scale of a few hundreds of kilometers, implying reduced gene flow among those fragmented habitats. 

Direct comparisons between our results and the empirical data in [100] are complicated by several factors. First, our study area straddled the boundary between and included parts of two of their regions (Upper Gila Mountains and Basin and Range West) which differed in apparent levels of gene flow and genetic structure. Second, our results demonstrate predicted future patterns given current landscape structure as a starting point, whereas results in Barrowclough et al. [100] were based on samples collected in the 1980s and 1990s and reflected genetic processes occurring in past landscapes. Third, sample sizes in [100] were relatively small and entirely lacking for most parts of our study area.

Despite these complicating factors, our results are at least partly consistent with empirical results. For example, under the IBD hypothesis, the Mogollon Rim area (corresponding to the Upper Gila Mountains Region in [100]) appears largely connected genetically, whereas the more highly fragmented area south of the Mogollon Rim (Basin and Range West region in Barrowclough et al. [100]) appears far less connected in terms of spatial gene flow (Figure 4 and Figure 5). Both results are largely consistent with empirical results in Barrowclough et al. [100].

In contrast, results under the IBR hypothesis (Figure 4 and Figure 5) generally show far less genetic connectivity than indicated by empirical results [100] across our entire study area and for both levels of fragmentation and levels of dispersal ability. There are several possible reasons for this apparent discrepancy. First, it may indicate that IBD is a better predictor of landscape connectivity in the Mexican spotted owl than IBR. That is, although this owl is highly selective for particular types of habitat for nesting, it appears capable of dispersing long distances across landscapes where such habitat is sparse or lacking to find suitable habitat patches. Alternatively, differences between our results and empirical data could be due to differences between past landscapes and resulting genetic processes and future processes given the current landscape. Finally, these differences may indicate differences in sensitivity between genetic assays used here and the metrics of genetic structure used in the previous empirical study, especially given their relatively low sample sizes and uneven spatial sampling [100]. Finally, it is possible that the effects of landscape resistance are more apparent at larger spatial scales than we examined and in more fragmented landscapes than our study area. At present, we are unable to distinguish between these hypotheses, which are not mutually exclusive. 

## 5. Conclusions

This simulation study of landscape genetics of the Mexican spotted owl provides an analytical framework to identify relationships between landscape connectivity and genetic divergence and develop a spatial model of gene flow. We also identified potential effects of fragmentation on genetic processes by comparing two empirical resistance models. Our approach is replicable and can be used to help understand landscape effects on gene flow and genetic diversity for other species. In addition, this approach has relatively small data requirements, which makes it especially useful for analyzing landscape genetics of rare species, such as the Mexican spotted owl in this study. The spatial model may be valuable in designing conservation and management plans and in guiding monitoring efforts. For example, our results suggest that habitat fragmentation/configuration may be as or more important than habitat amount in promoting gene flow. The current Recovery Plan for the Mexican spotted owl does not explicitly incorporate habitat configuration in management recommendations [42], but future plans may benefit from explicitly incorporating spatial pattern, including provision of larger areas of relatively high habitat concentration supporting multiple owl territories.

Our study approach can estimate variation in spatial patterns of genetic diversity and evaluate the effects of landscape features based on simulated genetic data but does not provide inferences regarding true genetic diversity and population structure. Rather, our results are useful for generating hypotheses. Those hypotheses cannot be directly tested at this time due to the limited empirical genetic data available and differences in time and spatial scales represented. Comparisons with the limited empirical genetic data suggest areas of both agreement and disagreement between those data and our results, however. Of particular importance, the empirical data suggests that, at the spatial scale and level of connectivity present in our study landscape, IBD may better describe spatial pattern in owl genetics than IBR. This is potentially an important finding with implications for understanding how dispersing owls perceive the landscape, but the strength of that conclusion is limited by the small samples and limited spatial coverage of existing genetic data for our study area. Comparisons with empirical data could be strengthened by gathering a more current, extensive, and spatially balanced sample of genetic material from owls within our study area. Better data on typical distances traveled and habitats used by dispersing owls also would aid in refining landscape simulation models.

Because transforming habitat suitability into landscape resistance carries uncertainty for many species [62,101], we also advocate for collection of more empirical genetic data to facilitate development of resistance models based on actual genetic differentiation [28,102,103]. Such models should improve our ability to simulate and understand genetic processes. We also recommend long-term monitoring and the collection of genetic data at the landscape level. Because of climate change and increasing habitat fragmentation, genetic connectivity and population structures are not likely to remain stationary. Data collected from these long-term monitoring efforts will provide important information for understanding potential spatiotemporal changes of genetic patterns in this species of concern. Because simulation studies do not replace but instead complement empirical studies, we encourage empirical genetic studies for cross-validation of results from simulation studies. Finally, information from this study should be interpreted cautiously and used as a general guide rather than for concise management prescriptions.

## Figures and Tables

**Table 1:** Mantel tests relating the proportion of shared alleles (D_ps_) and resistance distance matrices in the low-fragmentation model and the high-fragmentation model under high-dispersal (300 km) and low-dispersal (200 km) scenarios. Significance of matrix correlations was tested by 1000 permutations.

Model	Dispersal	Isolation	Mantel *r*	*p*
Low fragmentation	High	IBD	−0.800	<0.001
		IBR	−0.569	<0.001
	Low	IBD	−0.830	<0.001
		IBR	−0.402	<0.001
High fragmentation	High	IBD	−0.613	<0.001
		IBR	−0.648	<0.001
	Low	IBD	−0.646	<0.001
		IBR	−0.322	<0.001

## Supplementary Materials

The following are available online at http://www.mdpi.com/2073-4425/9/8/403/s1, Figure S1: Spatial patterns of observed heterozygosity, Figure S2: Spatial patterns of expected heterozygosity, Figure S3: Relationship between observed heterozygosity and landscape connectivity, Figure S4: Relationship between expected heterozygosity and landscape connectivity, Table S1: Relationship between D_ps_ and resistance distance, Table S2: Relationship between effective population size (Ns) and connectivity, Table S3: Relationship between allelic richness (A_r_) and connectivity, Table S4: Relationship between observed heterozygosity (H_o_) and connectivity, Table S5: Relationship between expected heterozygosity (H_e_) and connectivity.
