# Supplementary material for: Habitat Fragmentation Reduces Genetic Diversity and Connectivity of the Mexican Spotted Owl: A Simulation Study Using Empirical Resistance Models
Source: Genes (Basel). 2018 Aug 10;9(8):403. doi: 10.3390/genes9080403 (PMC6115790; doi:10.3390/genes9080403)
Supplement: Supplementary file 1 [file genes-09-00403-s001.pdf]

genes MDPI

Table S1. Relationship between D_{ps} and resistance distance. The highest R^2 of each scenario is highlighted in yellow.

Model	Dispersal	Isolation	Linear		Logarithmic		Exponential	
			R^2	*p*	R^2	*p*	R^2	*p*
Low fragmentation	High	IBD	0.640	<0.001	0.541	<0.001	0.642	<0.001
		IBR	0.324	<0.001	0.255	<0.001	0.005	<0.001
	Low	IBD	0.689	<0.001	0.571	<0.001	0.691	<0.001
		IBR	0.162	<0.001	0.161	<0.001	0.026	<0.001
High fragmentation	High	IBD	0.376	<0.001	0.353	<0.001	0.378	<0.001
		IBR	0.420	<0.001	0.500	<0.001	0.220	<0.001
	Low	IBD	0.418	<0.001	0.377	<0.001	0.421	<0.001
		IBR	0.104	<0.001	0.129	<0.001	0.034	<0.001

Table S2. Relationship between effective population size (N_s) and connectivity. The highest R^2 of each scenario is highlighted in yellow.

Model	Dispersal	Isolation	Linear		Logarithmic		Exponential	
			R^2	*p*	R^2	*p*	R^2	*p*
Low fragmentation	High	IBD	0.880	<0.001	0.732	<0.001	0.697	<0.001
		IBR	0.887	<0.001	0.809	<0.001	0.927	<0.001
	Low	IBD	0.800	<0.001	0.682	<0.001	0.616	<0.001
		IBR	0.932	<0.001	0.840	<0.001	0.762	<0.001
High fragmentation	High	IBD	0.330	<0.001	0.378	<0.001	0.311	<0.001
		IBR	0.670	<0.001	0.528	<0.001	0.586	<0.001
	Low	IBD	0.181	<0.001	0.238	<0.001	0.169	<0.001
		IBR	0.705	<0.001	0.592	<0.001	0.361	<0.001

Table S3. Relationship between allelic richness (A_r) and connectivity. The highest R^2 of each scenario is highlighted in yellow.

Model	Dispersal	Isolation	Linear		Logarithmic		Exponential	
			R^2	*p*	R^2	*p*	R^2	*p*
Low fragmentation	High	IBD	0.115	<0.001	0.021	<0.001	0.111	<0.001
		IBR	0.949	<0.001	0.930	<0.001	0.938	<0.001
	Low	IBD	0.061	<0.001	0.020	<0.001	0.058	<0.001
		IBR	0.849	<0.001	0.918	<0.001	0.256	<0.001
High fragmentation	High	IBD	0.077	<0.001	0.009	0.001	0.077	<0.001
		IBR	0.558	<0.001	0.670	<0.001	0.338	<0.001
	Low	IBD	0.108	<0.001	0.019	<0.001	0.107	<0.001
		IBR	0.476	<0.001	0.439	<0.001	0.227	<0.001

Table S4. Relationship between observed heterozygosity (H_o) and connectivity. The highest R^2 of each scenario is highlighted in yellow.

Model	Dispersal	Isolation	Linear		Logarithmic		Exponential	
			R^2	*p*	R^2	*p*	R^2	*p*
Low fragmentation	High	IBD	0.017	<0.001	0.008	0.003	0.016	<0.001
		IBR	0.949	<0.001	0.943	<0.001	0.945	<0.001
	Low	IBD	0.006	0.010	0.005	0.013	0.005	0.011
		IBR	0.631	<0.001	0.748	<0.001	0.125	<0.001
High fragmentation	High	IBD	0.038	<0.001	0.000	0.380	0.038	<0.001
		IBR	0.549	<0.001	0.656	<0.001	0.300	<0.001
	Low	IBD	0.109	<0.001	0.019	<0.001	0.109	<0.001
		IBR	0.397	<0.001	0.374	<0.001	0.204	<0.001

Table S5. Relationship between expected heterozygosity (H_e) and connectivity. The highest R^2 of each scenario is highlighted in yellow.

Model	Dispersal	Isolation	Linear		Logarithmic		Exponential	
			R^2	*p*	R^2	*p*	R^2	*p*
Low fragmentation	High	IBD	0.073	<0.001	0.007	0.004	0.072	<0.001
		IBR	0.936	<0.001	0.930	<0.001	0.929	<0.001
	Low	IBD	0.050	<0.001	0.016	<0.001	0.049	<0.001
		IBR	0.614	<0.001	0.731	<0.001	0.121	<0.001
High fragmentation	High	IBD	0.069	<0.001	0.007	0.005	0.069	<0.001
		IBR	0.525	<0.001	0.636	<0.001	0.282	<0.001
	Low	IBD	0.109	<0.001	0.018	<0.001	0.109	<0.001
		IBR	0.392	<0.001	0.369	<0.001	0.203	<0.001

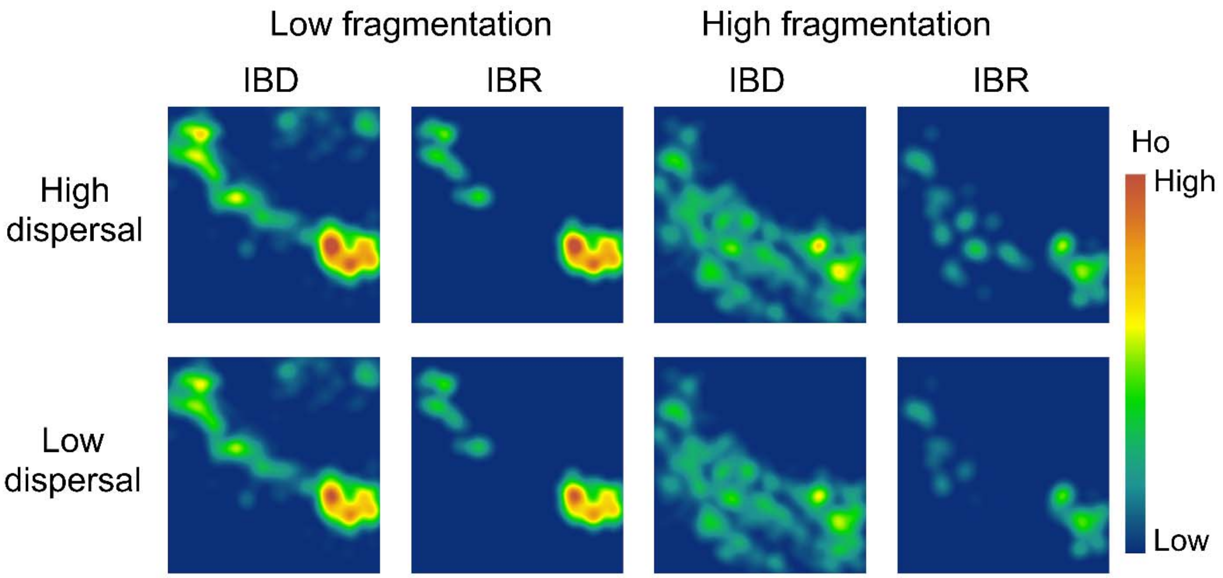

Figure S1. Spatial patterns of observed heterozygosity (H_o) in the low fragmentation model (left two columns) and the high fragmentation model (right two columns). IBD: isolation-by-distance. IBR: isolation-by-resistance. Top row: high dispersal scenario (300 km). Bottom row: low dispersal scenario (200 km).

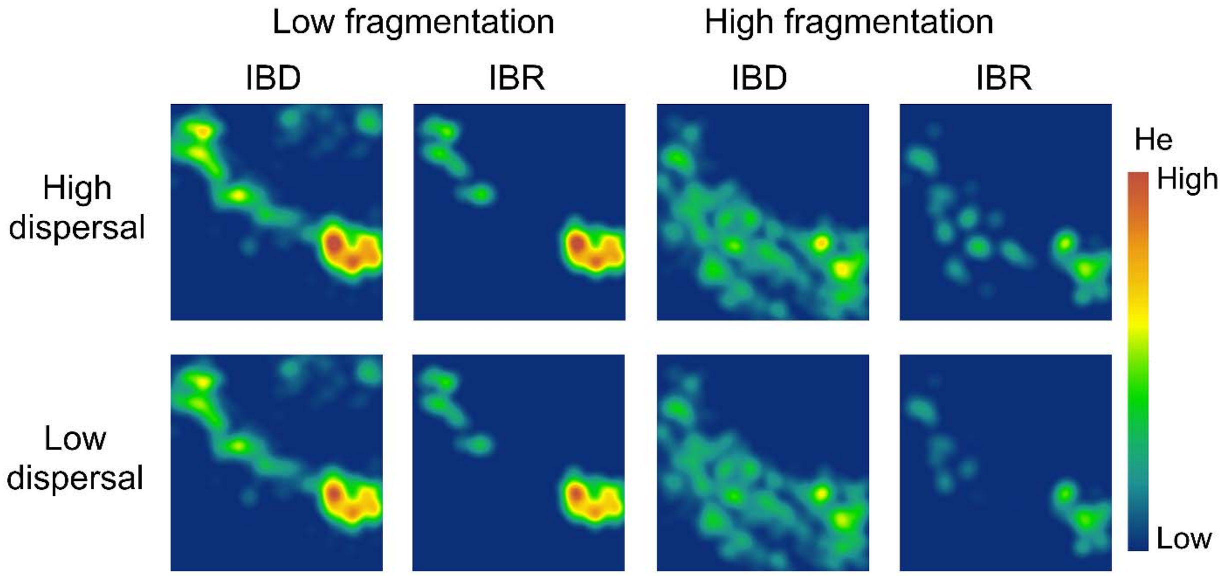

Figure S2. Spatial patterns of expected heterozygosity (H_e) in the low fragmentation model (left two columns) and the high fragmentation model (right two columns). IBD: isolation-by-distance. IBR: isolation-by-resistance. Top row: high dispersal scenario (300 km). Bottom row: low dispersal scenario (200 km).

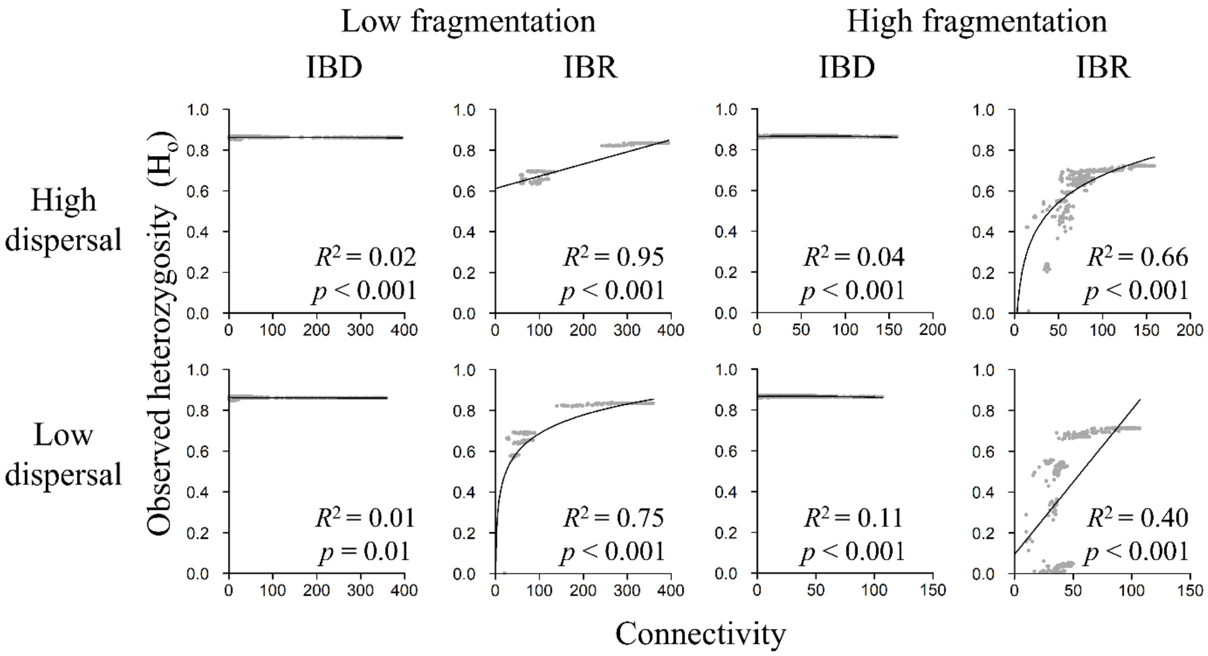

Figure S3. Relationship between observed heterozygosity (H_o) and landscape connectivity in the low fragmentation model (left two columns) and the high fragmentation model (right two columns). IBD: isolation-by-distance. IBR: isolation-by-resistance. Top row: high dispersal scenario (300 km). Bottom row: low dispersal scenario (200 km). Observed heterozygosity (y-axis) is calculated with software sGD. Connectivity (x-axis) is quantified in terms of cumulative resistant kernel density, representing the expected density of dispersing individuals. Gray markers represent the mean of 100 Monte Carlos simulation runs for each node. Adjusted R^2 and p-value are shown.

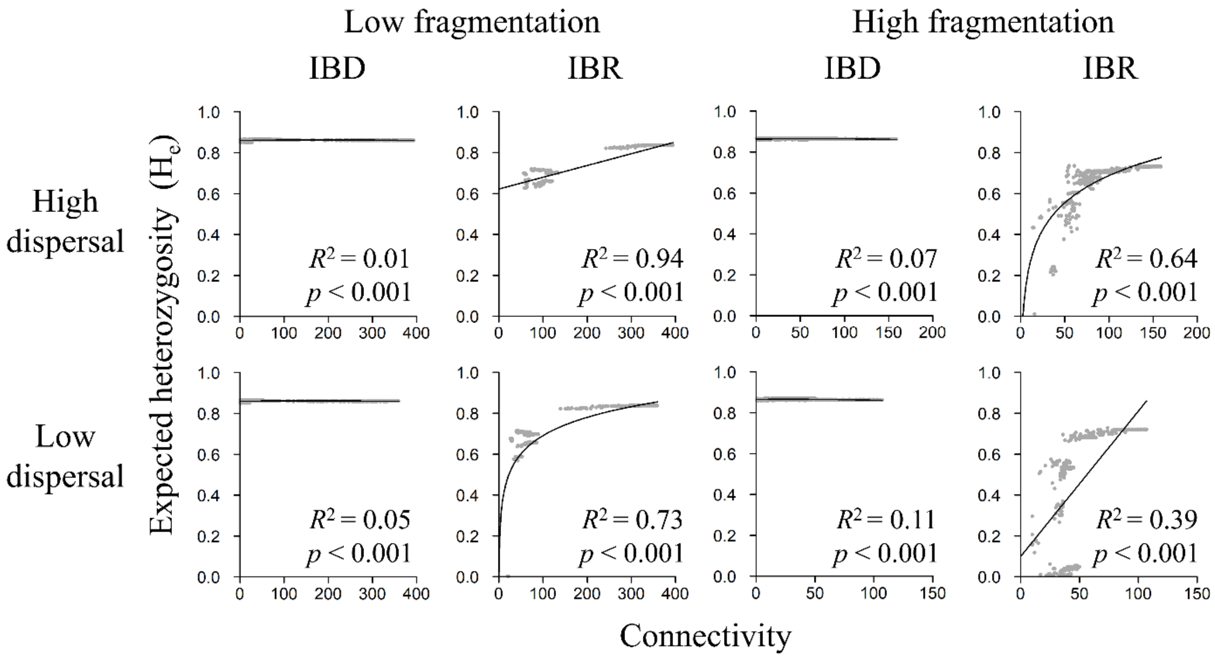

Figure S4. Relationship between expected heterozygosity (H_e) and landscape connectivity in the low fragmentation model (left two columns) and the high fragmentation model (right two columns). IBD: isolation-by-distance. IBR: isolation-by-resistance. Top row: high dispersal scenario (300 km). Bottom row: low dispersal scenario (200 km). Expected heterozygosity (y-axis) is calculated with software sGD. Connectivity (x-axis) is quantified in terms of cumulative resistant kernel density, representing the expected density of dispersing individuals. Gray markers represent the mean of 100 Monte Carlos simulation runs for each node. Adjusted R^2 and p-value are shown.
